# Transcriptome analysis of two radiated *Cycas* species and the subsequent species delimitation of the *Cycas taiwaniana* complex

**DOI:** 10.1002/aps3.11292

**Published:** 2019-10-16

**Authors:** Xin‐Hui Wang, Wei Wu, Shu‐Guang Jian

**Affiliations:** ^1^ Guangdong Provincial Key Laboratory of Applied Botany South China Botanical Garden Chinese Academy of Sciences Guangzhou 510650 People's Republic of China; ^2^ University of Chinese Academy of Sciences Beijing 100040 People's Republic of China

**Keywords:** comparative transcriptome, *Cycas*, gene duplication, positively selected genes, species delimitation

## Abstract

**Premise:**

*Cycas* is an important gymnosperm genus, and the most diverse of all cycad genera. The *C. taiwaniana* complex of species are morphologically similar and difficult to distinguish due to a lack of genomic resources.

**Methods:**

We characterized the transcriptomes of two closely related and endangered *Cycas* species endemic to Hainan, China: *C. hainanensis* and *C. changjiangensis*. Three single‐copy nuclear genes in the *C. taiwaniana* complex were sequenced based on these transcriptomes, enabling us to evaluate the species boundaries using the multispecies coalescent method implemented in the Bayesian Phylogenetics and Phylogeography program.

**Results:**

We obtained 68,184 and 81,561 unigenes for *C. changjiangensis* and *C. hainanensi*s, respectively. We identified six positively selected genes that are mainly involved in stimulus responses, suggesting that environmental adaptation may have played an important role in the relatively recent divergence of these species. The similar *K*_S_ distribution peaks at 1.0 observed for the paralogs in the two species indicate a common whole‐genome duplication event. Our species delimitation analysis indicated that the *C. taiwaniana* complex consists of three distinct species, which correspond to the previously reported morphological differences.

**Discussion:**

Our study provides valuable genetic resources for *Cycas* species and guidance for the taxonomic treatment of the *C. taiwaniana* complex, as well as new insights into evolution of species within *Cycas*.

The cycads, with their stout trunks and large, stiff evergreen leaves, were the dominant plant group in the Mesozoic Era (Martínez et al., 2017), with fossil cycads dating back to approximately 300 mya (Norstog and Nicholls, 1999). The cycads reached their greatest diversity during the Jurassic–Cretaceous, then shrank to approximately 300 species as the flowering plants began to flourish (Taylor et al., 2009); however, a radiation around 12 mya was revealed in recent studies (Nagalingum et al., 2011; Salas‐Leiva et al., 2013; Condamine et al., 2015).

Cycads belong to the cycad–ginkgo clade and are a sister group to other gymnosperms, meaning their study is informative for our understanding of seed plant evolution. In addition, the cycads are of great ornamental and economic value (Zheng et al., 2017; Liu et al., 2018). Although they survived three global extinction events in the past, cycads are now particularly vulnerable to anthropological activities, such as habitat loss and overexploitation. The IUCN Red List of Threatened Plants states that 12.5% of the world's vascular plants are threatened (Walter and Gillett, 1998); however, a staggering 82% of cycad species were included in the threatened categories (Raimondo and Donaldson, 2003). Roughly 40% of cycads grow in globally recognized biodiversity hotspots, leading some specialists to propose that they should be treated as flagship species for conservation (Zheng et al., 2017).

The cycads are currently assigned to two families: the Cycadaceae (containing one genus) and the Zamiaceae (comprising nine genera). These 10 genera contain about 353 species, which are primarily distributed in Asia, Africa, Australia, and South and Central America (Christenhusz et al., 2011). *Cycas* L. is the only genus in the Cycadaceae and consists of 116 species, making it the most diverse cycad genus. It is also the most widespread, being largely distributed across Asia and Australia, with a center of species diversity in China and Vietnam (Raimondo and Donaldson, 2003). *Cycas* species can be morphologically distinguished from other cycad species by their pinnule, which has a central midrib and lacks lateral veins, as well as their loosely aggregated, leaf‐like megasporophylls (Whitelock, 2002). *Cycas* is well supported as the sister to all of the other extant cycad genera (Liu et al., 2018). This genus was recently classified into six sections (Hill, 2008; Lindstrom et al., 2008), which is inconsistent with the previous infrageneric classification by De Laubenfels and Adema (1998), which divided *Cycas* into four subgenera containing 30 species. In addition, a few new species have been documented in recent years, and several species complexes were also proposed (Zhou et al., 2015; Singh, 2017), all of which further complicated the classifications within the genus. A comprehensive infrageneric classification incorporating both molecular phylogeny and morphological characters is therefore needed; however, the genetic diversity and classification of *Cycas* species remain unclear due to the lack of informative markers (Liu et al., 2018).

*Cycas hainanensis* C. J. Chen and *C. changjiangensis* N. Liu are two closely related species endemic to Hainan Island, China. These two species, together with four others (*C. taiwaniana* Carruthers, *C. fairylakea* D. Y. Wang, *C. szechuanensis* W. C. Cheng & L. K. Fu, and *C*. *lingshuigensis* G. A. Fu), constitute the *C. taiwaniana* complex. These six taxa are all endemic to southern China, and are mainly distributed across Hainan, Guangdong, and Fujian (Liu and Qin, 2004). These six taxa are morphologically similar and the relationship among them remains unclear; therefore, the *C. taiwaniana* complex is regarded as a taxonomically controversial group (Liu and Qin, 2004). In the *Flora of China* (Chen and Stevenson, 1999), *C. fairylakea* was considered a synonym of *C. taiwaniana*, and *C. hainanensis,* *C. changjiangensis*, and *C. szechuaniana* were listed as distinct taxa. Based on their morphological characteristics, Liu and Qin (2004) proposed the collapse of *C. hainanensis* and *C. changjiangensis* into *C. taiwaniana* and *C. fairylakea* into *C. szechuanensis*, such that the complex contained only two valid species (*C. taiwaniana* and *C. szechuanensis*). Using RAPD markers, Nong et al. (2011) recommended that *C. hainanensis*,* C. szechuanensis*,* C. fairylakea*, and *C. taiwaniana* were considered distinct species.

Species delimitation, such as the resolution of the *C. taiwaniana* complex, is essential for biogeography, conservation, macroevolution, and population genetic studies. Species are usually considered to be evolutionarily distinct lineages based on characteristics such as their monophyly, morphological differentiation, and reproductive isolation (Stockman and Bond, 2007; Fujita et al., 2012; McKay et al., 2013); however, some cryptic species (distinct species but incorrectly recognized as one species) (Bickford et al., 2007) are still difficult to resolve (Bickford et al., 2007; Struck et al., 2017; Moritz et al., 2018). Molecular genomic data are increasingly being used to clarify species boundaries (Kuchta et al., 2016; Hurtado‐Burillo et al., 2017). Recently, coalescent‐based species delimitation approaches using genetic markers have been developed to delimit species (Burbrink et al., 2011), while other researchers have considered the Bayesian Phylogenetics and Phylogeography (BPP) method, which implements highly parameterized models, to be more effective for species delimitation (Carstens et al., 2013; Luo et al., 2018). Based on the multispecies coalescent model, the BPP framework integrates uncertainty in gene trees to generate a robust species delimitation (Yang and Rannala, 2014; Yang, 2015). Empirical studies have revealed that BPP could generate delimitation results that were consistent with those of other widely used methods, such as the generalized mixed Yule‐coalescent and Poisson tree processes models (Previšić et al., 2016; Nieto‐Montes de Oca et al., 2017). BPP has been applied to delimit species within various species complexes and cryptic species groups (Mason et al., 2016; Moritz et al., 2018).

To date, the 1,000 Plants project has released the transcriptomes of two *Cycas* species, *C. micholitzii* and *C. rumphii* (Matasci et al., 2014), neither of which belongs to the *C. taiwaniana* complex. Transcriptomes are still unavailable for *C. hainanensis* and *C. changjiangensis*, which hindered our understanding of when and how these two endemic *Cycas* species diverged on Hainan Island. With the development of cost‐effective RNA‐Seq methods, transcriptome analyses have become a routine approach for studying non‐model organisms (Mao et al., 2016). RNA‐Seq has also been widely used in gene discovery, marker development, and the investigation of the evolution of polyploidy in plants (Feng et al., 2016a, b; Herraiz et al., 2016; Lin et al., 2016; Yessoufou et al., 2017).

In this study, we sequenced, de novo assembled, and annotated the transcriptomes of *C. hainanensis* and *C. changjiangensis*. We had three objectives: (1) characterize the transcriptomes of *C. hainanensis* and *C. changjiangensis*; (2) infer their whole‐genome duplication (WGD) events, estimate the divergence time between the two *Cycas* species, and identify positively selected genes to understand their adaptive evolutionary dynamics; and (3) evaluate the species boundaries and taxonomic status of these controversial taxa in the *C. taiwaniana* complex using a multispecies coalescent method based on single‐copy nuclear genes identified from our novel transcriptome data.

## MATERIALS AND METHODS

### Plant materials, RNA extraction, cDNA library preparation, and transcriptome sequencing

The six taxa of the *C. taiwaniana* complex were collected in the Hainan, Guangdong, and Fujian provinces of southern China. Young and healthy leaves were dried in silica gel immediately after collection for future use. Each sampled *C. hainanensis* and *C. changjiangensis* individual was transplanted in the South China Botanical Garden in Guangzhou.

Fresh leaves were collected, immediately frozen in liquid nitrogen, and stored at −80°C until required. The total RNA was isolated from each sample using the TRIzol Reagent (Thermo Fisher Scientific, Waltham, Massachusetts, USA), according to the manufacturer's instructions. The RNA was treated with RNase‐free DNase I (TaKaRa Bio Inc., Otsu, Shiga, Japan) to avoid genomic DNA contamination. The quality and quantity of the total RNA were determined using an Agilent 2100 Bioanalyzer (Agilent Technologies, Santa Clara, California, USA) and a Nanodrop ND1000 (Thermo Fisher Scientific), respectively. The cDNA libraries were constructed using a cDNA Synthesis kit (Illumina, San Diego, California, USA), then sequenced using 125‐bp paired‐end reads on the Illumina HiSeq 2500 platform at Berry Genomics, Beijing, China.

### De novo transcriptome assembly and functional annotation

After the adapters were removed, the raw reads for both species were subjected to de novo assembly using Trinity (trinityrnaseq‐; version 2.2.0) (Grabherr et al., 2011), and the data were cleaned using the trimmomatic subpackage with default settings (min_kmer_cov set to 2). Contigs longer than 300 bp were retained to be annotated. Unigenes for these contigs were obtained by the removal of redundant transcripts with an identity threshold of 0.90 using CD‐HIT version 4.6 (Fu et al., 2012). The completeness of the assemblies for the two species was evaluated using the software BUSCO version 2.0 (Simão et al., 2015), in which the unigenes were BLASTX searched against the 425 universal single‐copy eukaryote ortholog sets.

All unigenes were searched against the National Center for Biotechnology Information (NCBI) non‐redundant protein (Nr) database using a BLASTX search with an *E*‐value cutoff of 1.0E–6. Based on the best BLASTX hits from the Nr database, the Gene Ontology terms (GO) and the Kyoto Encyclopedia of Genes and Genomes (KEGG) pathways were retrieved using BLAST2GO with an *E*‐value cutoff of 1.0E–6 (Conesa et al., 2005). Finally, the distributions of the GO terms in the three categories (biological processes, molecular functions, and cellular components) were plotted using Web Gene Ontology Annotation Plot (Ye et al., 2006).

### Ortholog identification between *C. hainanensis* and *C. changjiangensis* and an adaptive loci screen

The protein‐coding regions and amino acid sequences were extracted using TransDecoder version 3.0.0 (http://transdecoder.sourceforge.net/). Based on the protein‐guided DNA alignments, putative orthologs between *C. hainanensis* and *C. changjiangensis* were identified by their reciprocal best BLAST hits (the *E*‐value cutoff was set at 1.0E–10 using a BLASTN with the reciprocal best matches [RBM] method; Li et al., 2003). The YN algorithm, implemented in *K*_A_ *K*_S_ Calculator version 1.2 (Zhang et al., 2006), was used to calculate the non‐synonymous substitution rate (*K*_A_), the synonymous substitution rate (*K*_S_), and the *K*_A_ */K*_S_ ratio for each pair of orthologs. After the removal of orthologs with a *K*_S_ > 2, the orthologous loci with *K*_A_/*K*_S_ ratios significantly larger than 1 (*P* value < 0.05) were considered to be candidates under positive selection.

### Inferring ancient WGDs based on paralog age distributions

Duplicated gene pairs and their divergence in terms of *K*_S_ were inferred using custom Perl scripts (Appendix S1) (Barker et al., 2008) in the following five steps: (1) identification of gene family members with at least a 40% sequence similarity over 300 bp using MegaBLAST; (2) extraction of reading frames according to the BLASTX hits against the predicted proteins (obtained by TransDecoder) using GeneWise version 2.2.2 (Birney et al., 2004), with a minimum cutoff of a 30% sequence similarity over at least 150 sites; (3) alignment of duplicate pairs using MUSCLE version 3.6 (Edgar, 2004); (4) calculation of *K*_S_ values for each duplicate pair using the maximum likelihood method implemented in codeml of the PAML package (Yang, 2007) under the F3‐4 model (Goldman and Yang, 1994); and (5) simple hierarchical clustering of each gene family to calculate the node *K*_S_ values. Gene pairs with *K*_S_ = 0 or *K*_S_ > 2 were removed to reduce the multiplicative effects of multicopy gene families and synonymous substitution saturations, respectively, on the *K*_S_ values (Vanneste et al., 2013). R (https://www.r-project.org/) was used to generate a density plot of the *K*_S_ values, which represented the age distributions of the paralogs. These age distributions can be used to identify ancient WGDs (Li et al., 2015).

### Mining of single‐copy nuclear genes and the Bayesian species delimitation analysis

Duarte et al. (2010) identified 959 sets of single‐copy nuclear genes shared by *Arabidopsis*,* Populus*,* Vitis*, and *Oryza* (known as the APVO genes). The protein sequences encoded by the APVO genes were obtained from the TAIR10 database (https://www.arabidopsis.org/) and queried against the orthologous EST database between *C. hainanensis* and *C. changjiangensis* using a TBLASTN search with an *E*‐value cutoff of 1.0E–10 (Altschul et al., 1997). The orthologous pairs with best hits in the two *Cycas* species were selected for the following primer design using SeqMan version 7.10 (DNASTAR Inc., Madison, Wisconsin, USA). The sequences were BLASTX searched against the *Arabidopsis thaliana* protein database with an identity threshold of 0.75. Exon‐anchoring and intron‐spanning primers for these sequences were designed using Primer Premier 5 (Premier Biosoft, Palo Alto, California, USA).

Eight to ten individuals from each of the six *C. taiwaniana* complex species were randomly selected for single‐copy nuclear gene sequencing. The sampling information used for the *C. taiwaniana* complex is summarized in Table 1. The total genomic DNA was extracted from each individual using the cetyltrimethylammonium bromide (CTAB) method (Doyle and Doyle, 1987). The PCR amplification was performed in a 20‐μL volume reaction using the following protocol: an initial denaturation at 95°C for 4 min; followed by 34 cycles of 95°C for 30 s, 55–58°C for 1 min, and 72°C for 1 min; with a final extension at 72°C for 10 min. The product sizes were determined on 1.5% agarose gels in TBE buffer, stained with ethidium bromide, then purified with a Gel Extraction Mini Kit (QIAGEN, Hilden, Germany). The PCR products were sequenced using an ABI 3730 DNA analyzer with a BigDye Terminator version 3.1 Cycle Sequencing Kit (Applied Biosystems). All generated sequences were deposited in GenBank under the accession numbers MH636113–MH636286. The sequences were aligned and edited using SeqMan version 7.10 (DNASTAR) for the following analysis.

**Table 1:** Details of sample locations and number of individuals surveyed for the single‐copy nuclear gene sequences of the *Cycas taiwaniana* complex.

Species name	Location	Latitude (°N)	Longitude (°E)	*n*
*C. hainanensis*	Lingshui, Hainan	18.667	109.933	10
*C. changjiangensis*	Changjianglizu, Hainan	19.000	109.117	8
*C. lingshuigensis*	Lingshui, Hainan	18.700	109.833	10
*C. szechuanensis*	Guangzhou, Guangdong	23.183	113.383	10
*C. taiwaniana*	Zhangzhou, Fujian	23.967	117.283	10
*C. fairylakea*	Futian, Shenzhen	22.567	114.083	10
Total				58

Note

*n* = number of individuals sampled.

The species delimitation for the *C. taiwaniana* complex was implemented using BPP version 3.3 (Yang, 2015) based on the three single‐copy nuclear genes. The BPP program uses reversible‐jump Markov chain Monte Carlo (rjMCMC; Yang and Rannala, 2014) to estimate the posterior probabilities of potential species delimitations to guide the generation of species trees, taking into account uncertainties in the species phylogeny and incomplete lineage sorting (Kuchta et al., 2016). The guide tree containing all six species of the *C. taiwaniana* complex was generated based on the Bayesian inference tree for the *Cycas* genus (Liu et al., 2018). The gamma prior G (2, 1000) was assigned to the population size parameters (θ), and the gamma prior G (2, 2000) was assigned to the root of the species tree (τ), which reflected a relatively small ancestral population and a shallow divergence. For the rjMCMC, after a burn‐in of 8000, the samples were collected every generation until 100,000 samples were obtained (108,000 iterations total). Each analysis was run at least twice to confirm the consistency among runs.

## RESULTS

### Transcriptome de novo assembly and functional annotation

After removing or trimming the low‐quality reads using the Trinity software, we obtained 26,702,049 clean reads for *C. changjiangensis* and 32,903,256 clean reads for *C. hainanensis* for the de novo assembly. A total of 91,603 contigs with an N50 length of 1119 bp for *C. changjiangensis* and 108,015 contigs with an N50 length of 1180 bp for *C. hainanensis* were also obtained (Table 2). All contigs were reassembled into 68,184 unigenes with a mean length of 777 bp for *C. changjiangensis*, and 81,561 unigenes with a mean length of 761 bp for *C. hainanensis* (Appendix S2). These numbers of unigenes were both higher than were previously obtained for *C. rumphii* (22,909) and *C. micholitzii* (54,202) (Matasci et al., 2014). An analysis using BUSCO revealed that the completeness of the assemblies was 58% (253/429) for *C. changjiangensis* and 65% (282/429) for *C. hainanensis*.

**Table 2:** Summary of transcriptome assemblies for *Cycas changjiangensis* and *C. hainanensis*.

Assembly results	*C. changjiangensis*	*C. hainanensis*
Transcripts		
Number	91,603	108,015
N50 (bp)	1119	1180
GC percentage (%)	46	46
Average length (bp)	837.58	837.96
Maximum length (bp)	12,990	14,608
Unigenes		
Number	68,184	81,561
N50 (bp)	996	983
Average length (bp)	777.49	760.79
Maximum length (bp)	12,990	14,608

**Figure 1:**
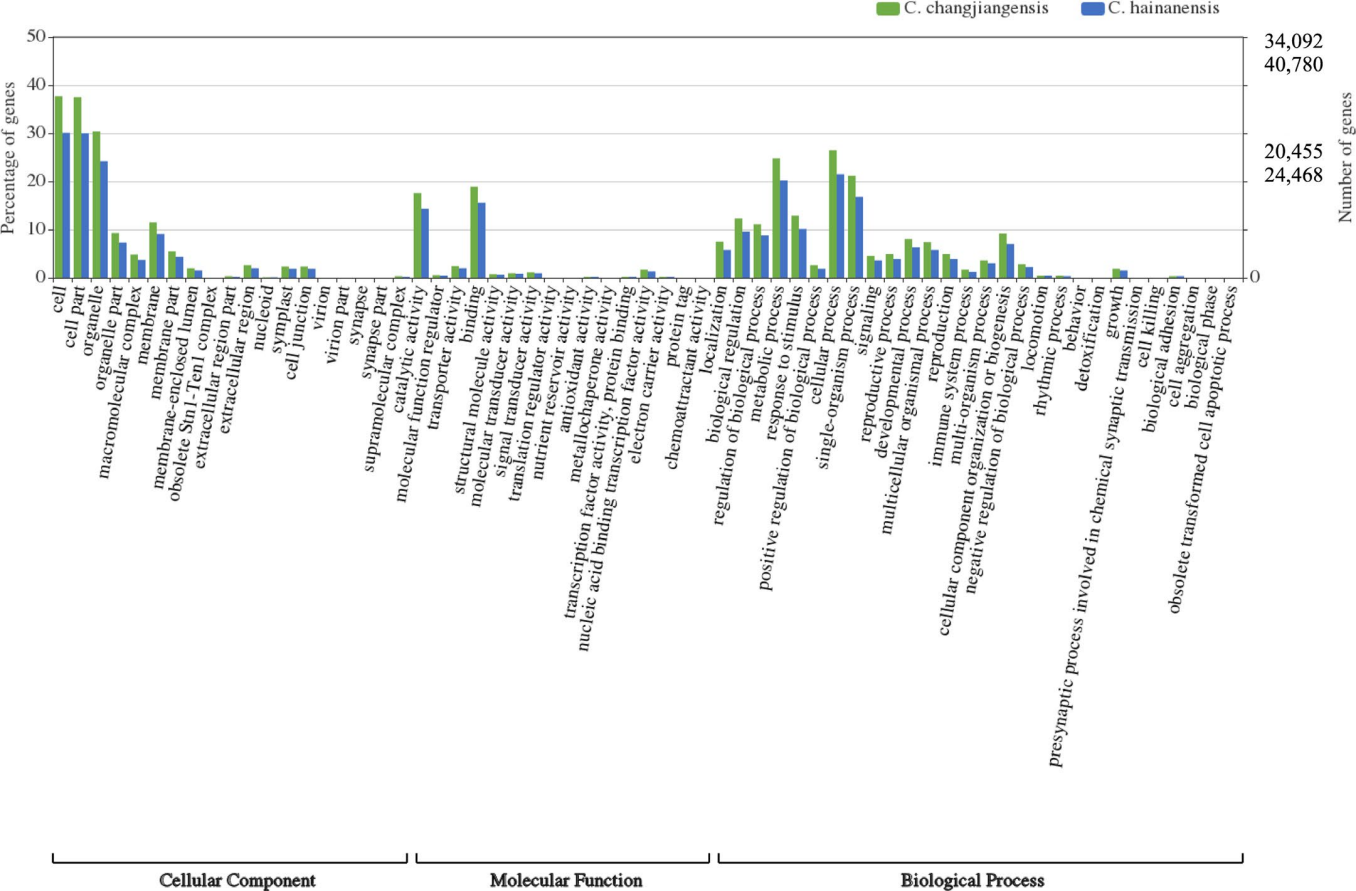
Comparison of GO term distributions between the two *Cycas* species. *Cycas changjiangensis* had 40,780 transcripts and 24,468 unigenes annotated to GO terms, and *C. hainanensis* had 34,092 transcripts and 20,455 unigenes annotated to GO terms.

The *C. changjiangensis* unigenes were annotated using the public databases as follows: 35,512 (52%) were annotated using the Nr database, 32,877 (48%) using the Eukaryote Orthologous Groups (KOG) database, and 19,086 (28%) were annotated with KEGG functional pathways (Table 3). The *C. hainanensis* unigenes were annotated using the public databases as follows: 36,390 (45%) were annotated using the Nr database, 34,134 (42%) using the KOG database, and 19,127 (23%) using the KEGG functional pathways. A total of 34,092 (50%) and 20,455 (25%) *C. changjiangensis* and *C. hainanensis* unigenes, respectively, were assigned to at least one GO term. In the biological process category, terms related to cellular, metabolic, and single‐organism processes were the most enriched (Fig. 1). In the cellular component category, the cell and organelle functions were most prominent, whereas in the molecular function category, the two major GO terms were binding and catalytic activity. These categories were similarly distributed in both *Cycas* species.

**Table 3:** Summary of annotation percentages of the *Cycas changjiangensis* and *C. hainanensis* unigenes using their similarity to annotated sequences in public databases.

Database	*C. changjiangensis*		*C. hainanensis*	
	No. of unigenes	Annotation percentage (%)	No. of unigenes	Annotation percentage (%)
Nr	35,512	52	36,390	45
KOG	32,877	48	34,134	42
KEGG	19,086	28	19,127	23
GO	34,092	44	20,455	38
Total number of annotated unigenes	68,184		81,561	

Note

GO = Gene Ontology database; KEGG = Kyoto Encyclopedia of Genes and Genomes; KOG = Eukaryote Orthologous Groups database; Nr = NCBI non‐redundant protein database.

### Ortholog divergences and ancient polyploidy

**Figure 2:**
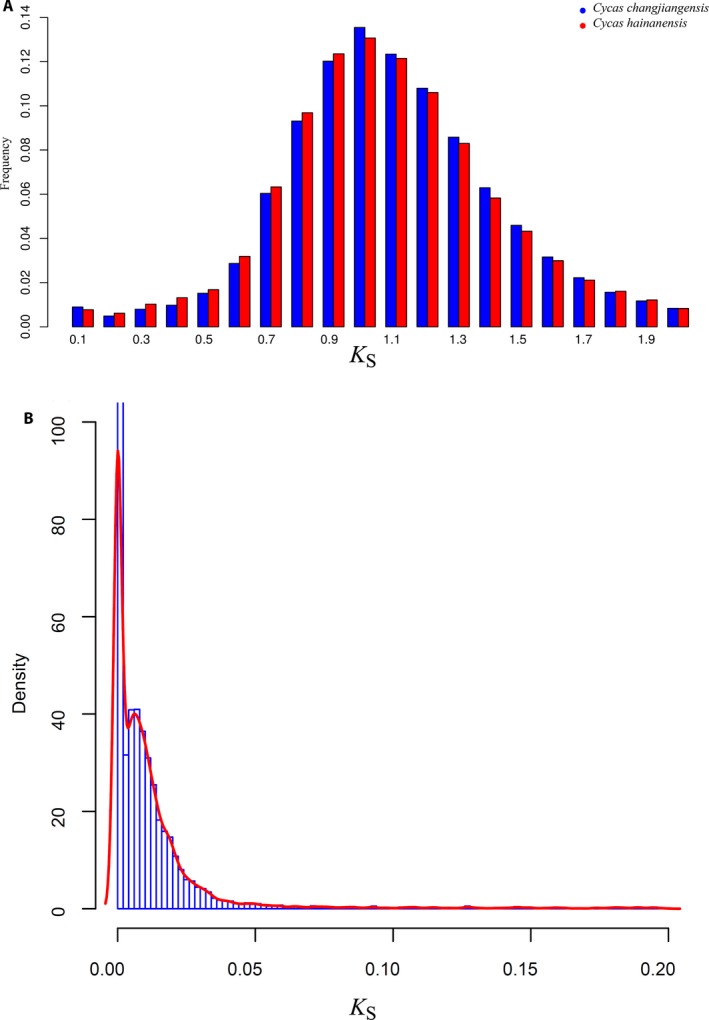
Divergence time and whole‐genome duplication in *Cycas changjiangensis* and *C. hainanensis*. (A) Frequency distribution of the *K*_S_ values for the paralogs in *C. changjiangensis* and *C. hainanensis*. The peaks indicate the ancient whole‐genome duplication event. (B) Age density distribution of orthologs between *C. changjiangensis* and *C. hainanensis*. The peak indicates the speciation event.

We identified 109,782 and 91,575 paralogous gene pairs with a *K*_S_ ≤ 2 in *C. hainanensis* and *C. changjiangensis*, respectively. The frequency of the *K*_S_ of the intragenomic paralog distribution peaked at 1.00 for *C. hainanensis* and 1.01 for *C. changjiangensis* (Fig. 2A). A conifer‐corrected synonymous substitution rate of 0.68 × 10^−9^ synonymous substitutions per site per year (Buschiazzo et al., 2012) was used to calculate the implied WGD event. This rate was used because the rate in cycads is not available; the conifers, as a closely related species to the cycads, provide a reasonable alternate substitution rate. Using this synonymous substitution rate, the implied WGD event was approximately 763 mya when calculated using the formula T = K / (2*r), where ‘K’ denotes the synonymous substitutions and ‘r’ denotes the substitution rate.

Using the reciprocal best hit method with the BLASTN algorithm, we also identified 10,195 orthologs between *C. hainanensis* and *C. changjiangensis*. Assuming that *K*_S_ occurs at a constant mutation rate over time, this value can be regarded as an indicator of the age of the duplicated genes. For each pair of orthologs, we estimated the *K*_S_ between the two species. The peak of the *K*_S_ values between *C. hainanensis* and *C. changjiangensis* was 0.0062 ± 0.0017 (Fig. 2B), suggesting a minor divergence between these two species. Using the same conifer‐corrected synonymous mutation rate of 0.68 × 10^–9^ per site per year (Buschiazzo et al., 2012), the divergence of these species was dated at approximately 4.6 mya.

### Positively selected genes and adaptive evolution

**Figure 3:**
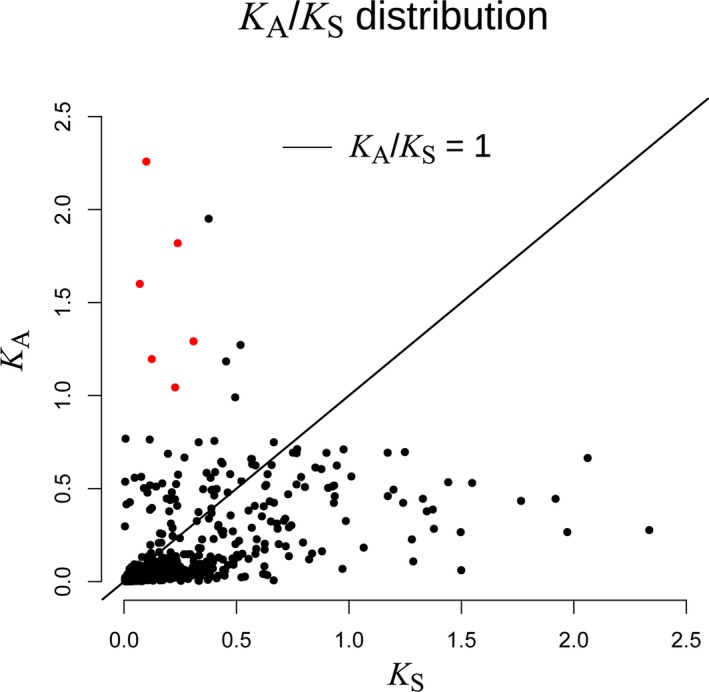
*K*_A_/*K*_S_ distribution of orthologs between *Cycas hainanensis* and *C. changjiangensis*. The solid line marks *K*_A_/*K*_S_ = 1, and the red dots mark the genes with a *K*_A_/*K*_S_ ratio significantly greater than 1.

We calculated and plotted the non‐synonymous to synonymous substitution ratio (*K*_A_ */K*_S_) for the 10,195 pairs of orthologs between *C. hainanensis* and *C. changjiangensis* (Fig. 3). Among them, 468 pairs were identical, 5049 pairs had either synonymous (*K*_S_) or non‐synonymous (*K*_A_) substitutions, and 4678 pairs had both *K*_A_ and *K*_S_ substitutions. Only six pairs were found to have a *K*_A_ */K*_S_ ratio significantly greater than 1 when *K*_S_ ≠ 0 (Fig. 3, Appendix S3). According to their GO annotations, five of these six genes were assigned to metabolic processes, while the other was associated with a cellular process. Two of the six genes were annotated as being involved in stimulus responses (Appendix S3), while one (*Cch_25724*) was involved in the response to high light intensity and UV‐A. Another gene (*Cch_57817*) was involved in the response to salt stress and osmotic stress.

### Bayesian species delimitation

Of the 959 APVO genes, 817 were found have orthologous unigenes in both *C. hainanensis* and *C. changjiangensis* (Appendix S4). We selected four primer pairs to validate, and of these, three were able to successfully amplify sequences derived from the unigenes of both *C. hainanensis* and *C. changjiangensis* (UniGene_23309/UniGene_22424, UniGene_23322/UniGene_57421, and UniGene_62177/UniGene_10198) in all *Cycas* species. The primers used in the following BPP analysis are presented in Appendix S5.

**Figure 4:**
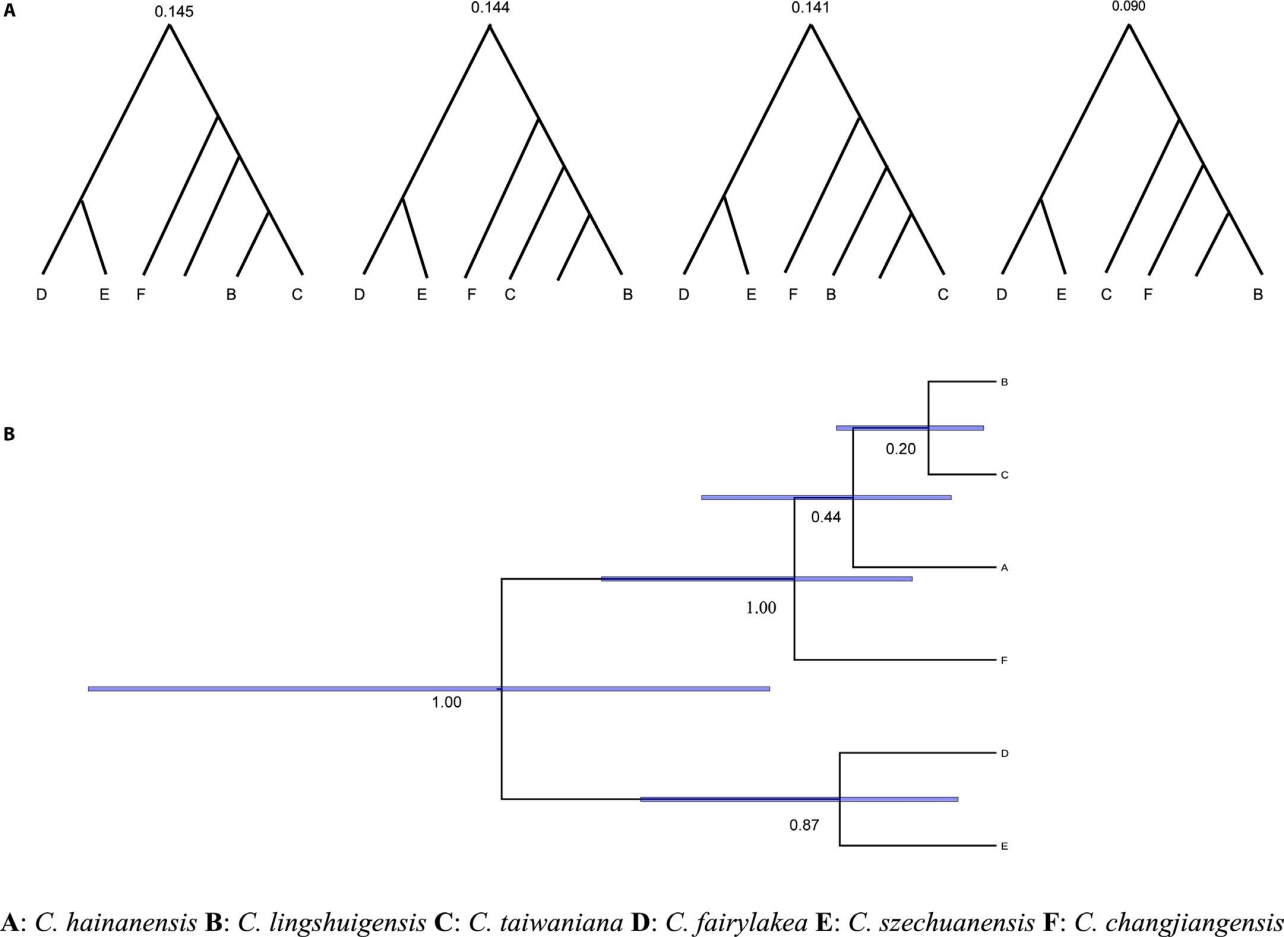
Bayesian species delimitation results for the *Cycas taiwaniana* complex. (A) The best four species trees and their posterior probabilities, with a total probability of 0.52. (B) A four‐lineage model with the highest posterior probability. The blue bar at each node marks 95% of the highest posterior density interval. The leftmost tree in A is used as the best guide tree in B. The value below the bar represents the posterior probability of a speciation event, and a threshold posterior probability of 0.95 is required to retain this node. The tree was drawn with FIGTREE using the BPP output.

The raw sequences were manually edited and aligned using SeqMan. The sequences of the three selected single‐copy nuclear genes (Gene001, Gene002, and Gene003) were 1344 bp, 1156 bp, and 1157 bp long after the alignment, respectively. Only individuals that yielded DNA sequences for all three single‐copy nuclear genes were used in the BPP analysis. Only those taxa with a posterior probability of speciation over a 95% threshold were considered distinct species. The Bayesian species tree estimation yielded 15 distinct trees in total, and a single best tree with a posterior probability of 0.145 (Fig. 4A). Using the guide tree and the rjMCMC algorithm, the posterior probabilities of different models and the posterior distribution of the parameters *τ* and *θ* were calculated for each model. The four‐lineage model was found to give a maximum posterior probability of 0.46 (six‐lineage model: 0.19, five‐lineage model: 0.22, three‐lineage model: 0.10, others omitted). For the four‐lineage model, three putative species (*C. hainanensis*,* C. taiwaniana*, and *C*. *lingshuigensis*) were combined into a single species (Fig. 4B), which was placed under the earliest proposed species *C. taiwaniana*. For the ancestral node of *C. fairylakea* and *C. szechuanensis*, a speciation posterior probability of 0.87 (below 0.95) indicated that the two lineages should be merged into one. In summary, our species delimitation analysis supported the existence of three true species within the *C. taiwaniana* complex (*C. changjiangensis*,* C. fairylakea* + *C. szechuanensis*, and *C. hainanensis* + *C. taiwaniana* + *C*. *lingshuigensis*).

## DISCUSSION

### Comparative transcriptome analyses provide new insights into the *Cycas* genus

Illumina‐based transcriptomic sequencing and de novo assembly provide a cost‐effective way of obtaining a large number of molecular markers for non‐model plants. Unfortunately, few transcriptomic studies and genomic assemblies are available for *Cycas* species, hampering evolutionary research into this genus, including studies of its divergence, speciation, and the identification of genes under positive selection. The unigenes and annotations identified in the present study will therefore be useful for further genome sequencing in *Cycas*.

The overall annotation rates for the two *Cycas* species studied here, *C. hainanensis* and *C. changjiangensis*, were not very high, with only about half of the unigenes annotated using the Nr (45% in *C. hainanensis*, 52% in *C. changjiangensis*) and KOG (42% in *C. hainanensis*, 48% in *C. changjiangensis*) databases. This may be due to the low representation of gymnosperm species in the available annotation databases (Li et al., 2015), although a small proportion of unannotated genes might be *Cycas*‐specific genes associated with certain genus‐specific characteristics (Mao et al., 2016; Guo et al., 2017). The high level of similarity between the GO categories associated with the *C. changjiangensis* and *C. hainanensis* unigenes might suggest a conserved gene repertoire for these species, and also indicates their very recent divergence (Yang et al., 2015).

A peak in the *K*_S_ distribution of orthologs between closely related species can facilitate the inference of speciation events (Blanc and Wolfe, 2004). The peak *K*_S_ between *C. hainanensis* and *C. changjiangensis* was 0.0062, suggesting that they are very closely related. By comparison, previous research found that the mean *K*_S_ values between congeneric species in several model plants were 0.03–0.10 (Zhang et al., 2013). We estimated that *C. changjiangensis* and *C. hainanensis* diverged approximately 4.6 mya, in the Middle Pliocene. The Plio‐Pleistocene global climate change is believed to have had an important influence on local habitats and the evolution of extant species (Dorsey et al., 2018; Zhang et al., 2019). In contrast with the conventional view of cycads as living fossils (Norstog and Nicholls, 1999), some studies have revealed that frequent speciation events have occurred in this group in the recent past (i.e., approximately 12 mya) (Nagalingum et al., 2011; Salas‐Leiva et al., 2013; Dorsey et al., 2018). *Cycas hainanensis* and *C. changjiangensis* are restricted to Hainan; however, it is unclear whether the two species migrated to the island from the mainland or experienced in situ adaptive differentiation in response to the different habitats present on Hainan Island.

Ancient polyploidy or WGD is commonly detected in angiosperm lineages (Wood et al., 2009), and many angiosperm lineages have experienced additional rounds of genome duplication (Jiao et al., 2011). Although polyploidy is relatively rare among extant gymnosperms (Li et al., 2015), several instances of paleopolyploidy have been identified (Khoshoo, 1959; Scott et al., 2016; Šmarda et al., 2016). Only one WGD event was shared by all seed plants, but recent studies have revealed that some gymnosperm taxa might have undergone additional WGD events, such as that in *Ephedra* (Ickert‐Bond and Wojciechowski, 2004; Ickert‐Bond and Renner, 2016) and a few cupressoid conifers (Husband et al., 2013). In our study, the paralog age distribution for *C. hainanensis* and *C. changjiangensis* had a *K*_S_ peak of approximately 1.0. Considering the various substitution rates among different taxa, this might correspond to the same WGD in another gymnosperm species, *Welwitschia mirabilis*, with a *K*_S_ value of 1.05 (Li et al., 2015). As is the case for most other gymnosperms, only one WGD event occurred in the evolutionary history of *C. hainanensis* and *C. changjiangensis*. We calculated the time of the WGD event (763 mya) using a previously extrapolated conifer mutation rate of 6.8E–10 known to be appropriate for use in cycads (Buschiazzo et al., 2012). The date of 763 mya is consistent with a previous study placing the ancestral gymnosperm genome duplication at 515 to 735 mya (Guan et al., 2016). This date predates most available estimates for the age of the land plants, however (Fiz‐Palacios et al., 2011). A WGD event has been inferred to have occurred at the time of the divergence of all seed plants, approximately 341 mya (Jiao et al., 2011; Wan et al., 2018). In addition, according to the phylogenetic placements of WGDs and the existing estimates for the ages of gymnosperm lineages, the conifer WGDs occurred ca. 210 to 275 mya (Cupressaceae + Taxaceae) and ca. 200 to 342 mya (Pinaceae) (Lu et al., 2014). The estimated date of 763 mya is therefore two‐ or three‐fold more ancient than the origin of the gymnosperms. This inconsistency may have been caused by the inappropriate use of the conifer‐corrected substitution rate of 6.8E–10 synonymous substitutions per site per year for the analysis of *Cycas*, as substitution rates are known to vary among different taxa. This conifer‐specific nucleotide substitution rate reflects a dramatically slower evolutionary rate in the conifers than was previously reported for the angiosperms (Buschiazzo et al., 2012). Although the substitution rates of most gymnosperm taxa are low (De La Torre et al., 2017), it is possible that the evolutionary rate in *Cycas* may be faster than the conifers. A future estimation of the *Cycas* WGDs should take the uncertainty of the substitution rates into account by using more samples, and gene tree–based approaches should be considered for further increases in accuracy.

*Cycas hainanensis* grows well in thick forests with high temperatures and wet substrates, whereas *C. changjiangensis* is mainly distributed on bare rock in arid conditions. These differences in the microhabitats of these two *Cycas* species suggests that natural selection may be the major force that has driven their divergence and evolution (Guo et al., 2017). The pattern of selective pressures across a genome may therefore be useful for making inferences about adaptive evolution (Kunstner et al., 2010; Siol et al., 2010).

The ratio of non‐synonymous to synonymous substitution rates is considered a good indicator of the natural selection pressure at the sequence level (Zhang et al., 2013). In this study, six of the 10,195 pairs of orthologs between *C. hainanensis* and *C. changjiangensis* were found to be under strong positive selection. These genes functioned mainly in cellular and metabolic processes and stimulus responses, according to their GO annotations. We identified the orthologs of these six positively selected genes in *Arabidopsis*. The ortholog of Cch_57817 was annotated as a glycine‐rich RNA‐binding GRP1A‐like protein, which are known to regulate RNA metabolism at the post‐transcriptional level and play important roles in the responses to abiotic and biotic stresses (Zhu et al., 2013). Previous studies have demonstrated that the genes encoding the glycine‐rich RNA‐binding proteins are upregulated in response to osmotic stresses such as drought and salt (Kim et al., 2008; Ciuzan et al., 2015). Another gene, *Cch_25724*, was found to be a homolog of a gene encoding the thylakoid membrane‐localized protein stress‐enhanced protein 1 (SEP1), which is upregulated in response to high light intensity (Heddad and Adamska, 2000). These two positively selected genes might be associated with the adaptive evolution of *C. hainanensis* and *C. changjiangensis* under the high salinity, high temperature, and high UV radiation conditions on Hainan Island. In addition to the low annotation rate of the gymnosperms in the public databases, the small number of genes annotated as being positively selected might be a consequence of their very recent divergence, which may not have allowed enough time for natural selection to act in a significant manner.

### Implications of the Bayesian species delimitation

Species are fundamental units in biological research, and are defined on the basis of various criteria (De Gregori et al., 2007). Molecular approaches have been widely used for species delimitation (Feng et al., 2016a; Shi and Yang, 2018); however, the taxonomy of the six taxa in the *C. taiwaniana* complex remains controversial. Furthermore, species delimitation in recently diverged complexes is difficult using traditional phylogenetic approaches. Investigating species delimitation and understanding the associated evolutionary processes became more feasible with the advent of genome sequencing. Previous studies have shown that coalescent‐based methods make full use of information even when small data sets are used (Yang and Rannala, 2014, 2017). Using the multispecies coalescent model with a small number of loci, the BPP program can more accurately identify species than other molecular methods, such as the Yule‐coalescent and Poisson tree processes models (Luo et al., 2018).

In this study, the BPP analysis recognized three species in the *C. taiwaniana* complex (*C. changjiangensis*,* C. fairylakea* + *C. szechuanensis*, and *C. hainanensis* + *C. taiwaniana* + *C*. *lingshuigensis*). Our species delimitation for the *C. taiwaniana* complex was in accordance with the previous morphological distinctions (Chen and Stevenson, 1999; Liu and Qin, 2004) and the phylogeny of *Cycas* reconstructed by Liu et al. (2018). Although only one wild individual was found in Fujian, Wang (1996) stated that *C. taiwaniana* also originates from Hainan Island and may possibly be synonymous with *C. hainanensis*. The megasporophylls of *C. hainanensis* fall within the range of morphological variation of those observed in *C. taiwaniana*. Furthermore, *C. lingshuigensis* and *C. hainanensis* are extremely morphologically similar, and are both distributed on Hainan Island. The lack of support for the distinction of *C. hainanensis*,* C. taiwaniana*,* C. lingshuigensis* (posterior probability = 0.26 and 0.43) may indicate their very recent divergence (Fig. 4). *Cycas changjiangensis* is morphologically distinct from the other taxa in the *C. taiwaniana* complex because of its enlarged stem base. *Cycas szechuanensis* and *C. fairylakea* are distinguished from the other taxa in the complex by their loose megasporophyll cones and the densely dark brown tomentum on their young leaves.

Other species delimitation methods use DNA data, such as the construction of haplotype phylogenetic trees, population clustering, and Bayes Factor Delimitation (Wiens and Penkrot, 2002; Luo et al., 2018). Using multiple methods (i.e., multiple‐coalescent species delimitation, population clustering, and phylogenetic analysis) to determine the species boundaries in future studies will result in more comprehensive insights regarding their delimitation. Species delimitation based on molecular approaches does not appeal to an explicit species concept, but rather can be used to build a taxonomic scheme for a set of samples (Mallo and Posada, 2016). Accordingly, additional evidence from species ecology, behavioral traits, geographic distributions, and population genetic analyses should be incorporated into the species delimitation framework.

## CONCLUSIONS

In this study, we characterized the transcriptomes of two *Cycas* species and obtained new genomic resources for these endangered plants. In addition, we identified three positively selected single‐copy nuclear genes that could be used to evaluate the species boundaries for the *C. taiwaniana* complex using a BPP analysis. Three distinct lineages were recognized, which will enhance our understanding of the evolution of these *Cycas* species.

## AUTHOR CONTRIBUTIONS

S.J. designed the research and collected the study materials. X.W. and W.W. performed the transcriptome and data analyses. X.W., W.W., and S.J. wrote the manuscript and made the revisions. All authors approved the final manuscript.

## Supporting information


**APPENDIX S1.** Custom Perl script.

**APPENDIX S2.** Length distribution of unigenes for (A) *Cycas changjiangensis* and (B) *C. hainanensis*.

**APPENDIX S3.** Annotation of six positively selected genes.

**APPENDIX S4.** Identification of *Cycas*‐specific single‐copy nuclear genes homologous to the APVO gene sets. The locus ID in *Arabidopsis* and counts for each unigene in both *C. changjiangensis* and *C. hainanensis* are provided.

**APPENDIX S5.** Information and amplification results for the single‐copy nuclear genes.

## Data Availability

The data sets analyzed during the current study are available from the corresponding author. Raw reads for *C. hainanensis* and *C. changjiangensis* were deposited in the National Center for Biotechnology Information (NCBI) Sequence Read Archive under the accession numbers SRR5438344 (*C. hainanensis*) and SRR5438343 (*C. changjiangensis*), respectively. The data sets, assembled transcriptomes, the GO term annotations, and orthologs are available in the Dryad Digital Repository (https://datadryad.org/review?doi=doi:10.5061/dryad.hs561q1; Wang et al., 2019). The DNA sequences have been deposited in GenBank (accessions MH636113–MH636286).
