# Supplementary material for: Transcriptome analysis of two radiated Cycas species and the subsequent species delimitation of the Cycas taiwaniana complex
Source: Appl Plant Sci. 2019 Oct 16;7(10):e11292. doi: 10.1002/aps3.11292 (PMC6814181; doi:10.1002/aps3.11292)
Supplement: Supplementary file 1 — APPENDIX S1. Custom Perl script. [file APS3-7-e11292-s001.docx]

**APPENDIX S1.** Custom Perl script.

#!usr/bin/perl -w

use strict;

die "USAGE :$0 <input_pro> <input_parapair> <pairs_kaks>" unless (@ARGV==3);

open(PRO,"<$ARGV[0]")||die "can't open protein sequences file1 $!";

open(FAS,"<$ARGV[1]")||die "can't open inputfile $!";

open(OUT,">>$ARGV[2]")||die "can't open outputfile $!";

my %pro;

my $seq;

my $id;

while(<PRO>){

chomp $_;

if(/\>(.*)\|(.*)\|(.*)\|(.*)\|(.*)/){

$id=$1;

$seq=();

}

else{

$seq.=$_;

$pro{$id}=$seq;

}

}

my $i=1;

my @block;

while(<FAS>){

chomp $_;

push @block,$_;

my $seqname1;

my $seqname2;

my $proseq1;

my $proseq2;

if($i%4==0){

$seqname1=(split/\>/,$block[0])[1];

$seqname2=(split/\>/,$block[2])[1];

if(exists $pro{$seqname1}){

$proseq1=$pro{$seqname1};

$proseq2=$pro{$seqname2};

push my @gene1,($block[0],$block[1]);

push my @protein1,($block[0],$proseq1);

push my @gene2,($block[2],$block[3]);

push my @protein2,($block[2],$proseq2);

open(PROTEIN1,">protein1.pep") || die "can't open outputfile $!";

open(GENE1,">gene1.cds")||die "can't open outputfile $!";

open(PROTEIN2,">protein2.pep") || die "can't open outputfile $!";

open(GENE2,">gene2.cds")||die "can't open outputifle $!";

print PROTEIN1 ($_,"\n") for @protein1;

print PROTEIN2 ($_,"\n") for @protein2;

print GENE1 ($_,"\n") for @gene1;

print GENE2 ($_,"\n") for @gene2;

close PROTEIN1;

close PROTEIN2;

close GENE1;

close GENE2;

my $genewise1="genewise protein1.pep gene1.cds -sum > res1.txt";

my $genewise2="genewise protein2.pep gene2.cds -sum > res2.txt";

system($genewise1);

system($genewise2);

open (RES1,"<res1.txt")||die "$!";

open (RES2,"<res2.txt")||die "$!";

my $seq1;

my $seq2;

my @info1;

my @info2;

while(<RES1>){

chomp $_;

push @info1,$_;

}

close RES1;

my @items1=split/\s+/,$info1[1];

$seq1=substr($block[1], $items1[5]-1,$items1[6]-$items1[5]+1);

@info1=();

@items1=();

while(<RES2>){

chomp $_;

push @info2,$_;

}

close RES2;

my @items2=split/\s+/,$info2[1];

$seq2=substr($block[3], $items2[5]-1,$items2[6]-$items2[5]+1);

@info2=();

@items2=();

push my @prank,($block[0],$seq1,$block[2],$seq2);

open (PRANK,">pairsseqs.txt")||die "$!";

print PRANK ($_,"\n") for @prank;

my $paraprank="./prank -d=pairsseqs.txt -o=pairseq -f=paml";

system($paraprank);

my $paracodeml="codeml";

system($paracodeml);

my $size;

my $filter;

my @filter;

open(FILTER,"<pairseq.best.phy")||die "$!";

while(<FILTER>){

chomp $_;

push @filter,$_;

}

$filter=shift @filter;

$size=(split/ /,$filter)[1];

if($size>=150){

open(RES,"<pamlres")||die "$!";

my @pamlres;

my $pamlres;

while(<RES>){

chomp $_;

push @pamlres,$_;

}

close RES;

$pamlres=pop @pamlres;

print OUT "$seqname1\t$seqname2\t$pamlres\n";

@pamlres=();

}

@protein1=();

@gene1=();

@protein2=();

@prank=();

@filter=();

}

@block=();

}

$i++;

}

close PRO;

close FAS;

close OUT;
