# Supplementary figures and images for: Transcriptome analysis of two radiated Cycas species and the subsequent species delimitation of the Cycas taiwaniana complex
Source: Appl Plant Sci. 2019 Oct 16;7(10):e11292. doi: 10.1002/aps3.11292 (PMC6814181; doi:10.1002/aps3.11292)

**APPENDIX S2.** Length distribution of unigenes for (A) *Cycas changjiangensis* and (B) *C. hainanensis*.

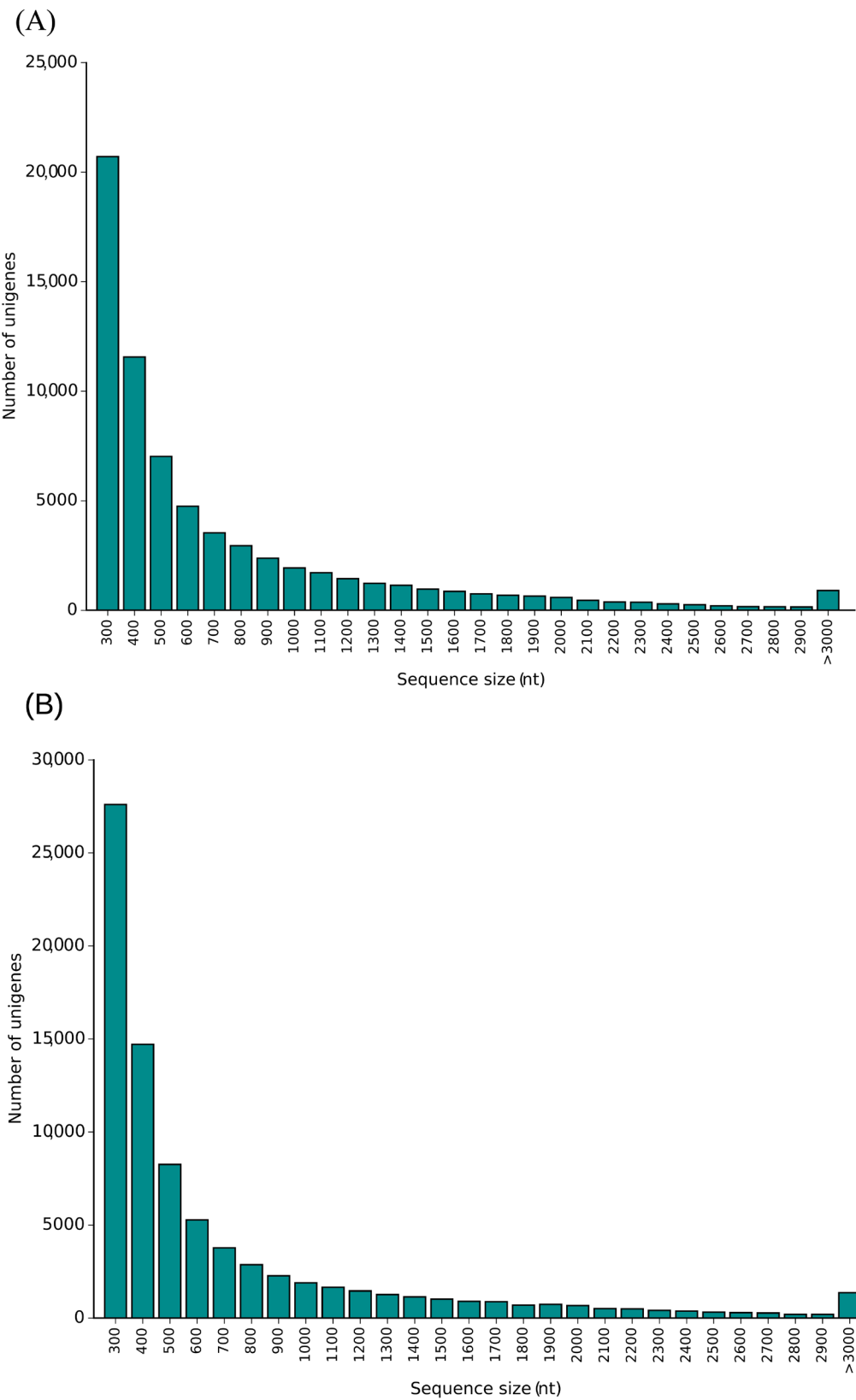

Supplement: Supplementary file 2 — APPENDIX S2. Length distribution of unigenes for (A) Cycas changjiangensis and (B) C. hainanensis. [file APS3-7-e11292-s002.pdf]
