# Supplementary material for: Transcriptome analysis of two radiated Cycas species and the subsequent species delimitation of the Cycas taiwaniana complex
Source: Appl Plant Sci. 2019 Oct 16;7(10):e11292. doi: 10.1002/aps3.11292 (PMC6814181; doi:10.1002/aps3.11292)
Supplement: Supplementary file 3 — APPENDIX S3. Annotation of six positively selected genes. [file APS3-7-e11292-s003.docx]

**APPENDIX S3.** Annotation of six positively selected genes.

| **Gene name** | ***K*_A_** | ***K*_S_** | ***K*_A_ / *K*_S_** | **GO annotation** |
| --- | --- | --- | --- | --- |
| *Cch_25724* (UniGene_25724) | 1.599 | 0.066 | 24.2 | Response to high light intensity/cellular response to UV-A |
| *Cch_1738* (UniGene_1738) | 1.195 | 0.119 | 10.0 | Metabolic process |
| *Cch_26849* (UniGene_26849) | 1.043 | 0.223 | 4.7 | Binding |
| *Cch_44433* (UniGene_44433) | 1.818 | 0.235 | 7.7 | Peptide receptor activity |
| *Cch_57817* (UniGene_57817) | 1.291 | 0.305 | 4.2 | Response to salt stress/cadmium ion/temperature stimulus |
| *Cch_61215* (UniGene_61215) | 2.257 | 0.095 | 23.7 | Cellular component |

*Note:* GO = Gene Ontology; *K*_A_ = non-synonymous substitution rate; *K*_S_ = synonymous substitution rate.
