# Supplementary material for: Transcriptome analysis of two radiated Cycas species and the subsequent species delimitation of the Cycas taiwaniana complex
Source: Appl Plant Sci. 2019 Oct 16;7(10):e11292. doi: 10.1002/aps3.11292 (PMC6814181; doi:10.1002/aps3.11292)
Supplement: Supplementary file 5 — APPENDIX S5. Information and amplification results for the single‐copy nuclear genes. [file APS3-7-e11292-s005.docx]

**APPENDIX 5.** Information and amplification results for the single-copy nuclear genes.

| **Locus** | **Unigenes** | **Primer sequences (5′–3′)** | ***T*_m_ (°C)** | **Expected product size (bp)** | **No. of amplified species** |
| --- | --- | --- | --- | --- | --- |
| Gene001 | UniGene_23309/UniGene_22424 | F: TCACCAGATTTGAAGATGGC  R: CCCCTCTTCAAATCGAGGTTG | 55 | 1069 | 6 |
| Gene002 | UniGene_23322/UniGene_57421 | F: CCAGTCTCCCAGTATCATGG  R: GCTGCATGATATTTCCAACC | 58 | 1106 | 6 |
| Gene003 | UniGene_62177/UniGene_10198 | F: TTTGGCACATAGGATACCCC  R: ATAGGATACCCCCACCAAGG | 56 | 1393 | 6 |

*Note:* *T*_m_ = melting temperature.
